# Differential Diagnosis Skills in Physician Assistant Students

**DOI:** 10.7759/cureus.83190

**Published:** 2025-04-29

**Authors:** Evan Leonard

**Affiliations:** 1 College of Health Professions and Medical Science, Barry University, Miami Shores, USA

**Keywords:** clinical skills, differential diagnosis, educational interventions, physician assistant education, team-based learning

## Abstract

This study explored physician assistant (PA) educators’ perceptions of a potential deficit in differential diagnosis proficiency (DDP) among PA students and sought to identify effective strategies for curricular improvement. DDP is a foundational clinical skill that requires robust medical knowledge, critical thinking, and the ability to synthesize patient information into diagnostic possibilities. Deficiencies in these skills can lead to diagnostic delays, unnecessary testing, and suboptimal patient outcomes. Despite its clinical importance, limited research has investigated how well PA programs cultivate DDP or what interventions may enhance it.

A cross-sectional, mixed-methods survey was administered to 68 PA educators via a secure listserv. The instrument included Likert-type questions and open-response items that assessed whether respondents perceived a deficit in DDP among their students, how severe they considered the issue to be, and what strategies they recommended for addressing it. Of the respondents, 64.7% (n = 44) affirmed the existence of a DDP deficit. The majority of those respondents (68.2%, n = 30) rated the issue as moderate in severity, while 13.6% (n = 6) considered it minor, and 18.2% (n = 8) deemed it significant.

When asked to recommend instructional strategies, respondents most frequently endorsed three equally preferred interventions: incorporating team-based learning events, utilizing instructor-led case studies, and integrating DDP content into existing medicine courses. Each of these received 23.3% of the total votes. Notably, team-based learning also emerged as the most commonly selected second-choice approach, suggesting strong overall support for active, collaborative learning formats. Respondents indicated that overburdened faculty and insufficient formal instruction were the most likely contributors to the DDP gap.

This study offers early evidence that PA educators widely perceive a need to enhance DDP training in existing curricula. The preference for interactive instructional strategies underscores a growing belief that passive, lecture-based methods may be insufficient for cultivating diagnostic reasoning skills. These findings support the integration of active learning modalities into PA education and lay the groundwork for further research. Future studies with larger, multi-institutional samples are needed to confirm these findings and evaluate the impact of specific interventions on DDP development and clinical performance.

## Introduction

Researchers have reported deficiencies in students of medical and physician assistant (PA) education programs [1]. The absence of skills can negatively impact the historical questioning and management of patients. In this study, the researcher proposes to determine whether physician assistant educators believe that there is an existing deficiency and whether adding content designed to help PA students develop differential diagnosis skills to the curriculum would benefit those students. There is a significant paucity of research regarding physician assistant students’ differential diagnosis skills.

Formulating an appropriate differential diagnosis is a crucial skill the diagnostician must be competent in in order to manage patients appropriately [1]. Formulating a differential diagnosis is where the art of medicine meets the science of medicine. Teaching how to formulate clinically relevant differential diagnoses is difficult. Graber et al discussed how poor medical students and other healthcare professionals were at formulating appropriate differential diagnoses [1]. One of the hypotheses for why clinical students were unable to formulate proficient differentials was that the diagnostician must first have extensive medical knowledge and then be able to synthesize the patient’s history and physical examination findings into possible disease processes. Clinical medicine students have an insufficient amount of medical knowledge when compared with practicing clinicians and were found to be inefficient at history taking and performing a physical examination [2]. Doctoral physical therapy students at Idaho State University participated in a study (n=27) measuring their confidence level in developing differential diagnoses for different musculoskeletal disorders. The researchers concluded, via post-course survey analysis, that the students’ confidence increased at the conclusion of the newly developed differential diagnosis course [3]. Fuentes researched whether pharmacy students would learn and benefit from an elective course in differential diagnosis. The post-course survey responses (n=21) demonstrated that the students thought they benefited significantly from the elective differential diagnosis course. The researchers created an elective course in differential diagnoses, which consisted of seven 2-hour class sessions. The students were assigned patient simulations and asked to come up with the correct primary and differential diagnoses. The students were assessed through one-on-one interactions with the faculty, and they had to present their cases to the faculty. Peer evaluations were also utilized. The result was that all 21 students were able to identify the correct diagnosis by the end of the course [4].

Differential diagnosis proficiency (DDP) requires that adequate patient data has been collected. Forming a differential diagnosis is critical to order appropriate diagnostics and management of a disease process. A survey of medical students showed that 72% of them felt the differential diagnosis assignments during their pathology course should be included in the course the following year. The students felt the assignments helped them improve their clinical decision-making skills. Eighty-six percent believed it helped them learn what questions to ask of the patients. The school also had students discuss and work through the patient encounter and differential diagnosis formulation. The authors concluded that the literature and their study further perpetuate the notion of how important good differential diagnosis is. They also concluded that incorporating differential diagnosis and clinical decision-making education within the basic science curriculum proved helpful [5]. 

The aim of this study is to explore the perceived deficiencies in differential diagnosis skills among PA students and to evaluate the effectiveness of proposed educational interventions.

## Materials and methods

The principal investigator (PI) conducted a cross-sectional survey study using a combination of Likert-type and open-response items to collect the responses of PA educators regarding: (a) Whether the respondents feel the clinical year students in PA programs in which the respondent teaches have the knowledge and skills to formulate differential diagnosis proficiency thoroughly, accurately, and reliably; (b) The extent the respondents feel the curricula in their institutions adequately teach differential diagnosis content, and (c) What they feel is the best way in which to integrate differential diagnosis content into their current curricula to develop their students’ knowledge and skills pertaining to differential diagnosis.

The PI pilot-tested the survey with doctoral-level classmates who are also educators in PA programs. The survey was developed by the PI with straightforward multiple-choice questions that were then validated by a team of five peers. The PI performed a literature review to identify the relevance and validity of the study. Participants were included if they were part-time or full-time faculty members or clinical preceptors providing instruction to students enrolled in a physician assistant program accredited by the Accreditation Review Commission on Education for the Physician Assistant (ARC-PA) within the United States. Individuals were excluded if they had never held a formal faculty position in such a program. Additional exclusion criteria were: those whose primary roles were administrative or support-based without instructional responsibilities, as well as physicians, nurse practitioners, or other healthcare professionals who served only in adjunct or guest lecturer capacities without formal PA faculty appointments. Individuals currently enrolled as PA students or those who served solely as clinical preceptors without academic titles were also excluded.

The participants were contacted via email to take part in the study. The email addresses were sourced from the Physician Assistant Education Association's (PAEA) listserv, which contains all current members' email addresses. Only former or current PA faculty members received an email. The timeline from the distribution of the emails to data collection spanned eight weeks. The potential participants received a website link to the survey. Informed consent was the first screen the participant saw. If the participants agreed to the terms, they proceeded to participate in the survey. If the participants decided not to participate, they exited to a “thank you” screen and did not reengage the survey at all. See Appendices for the survey questions. Incomplete or missing values were discarded and not used in the data analysis. 

The participants who agreed to participate accessed the survey, which began with a few items to solicit basic demographic information (e.g., how many years the participant has been involved in PA education, and whether the participant's primary instructional responsibilities were in the clinical or didactic phase of the PA students’ education). The PI collected the data via Surveymonkey.com (SurveyMonkey, San Mateo, USA) and used the intuitive, basic analytical tools available in SurveyMonkey to conduct the initial analyses of the results. The researcher sent three reminder emails over the course of 8 weeks.

After examining and cleaning the data, the PI imported the data into SPSS (IBM Corp., Armonk, USA) for final analysis. Kendall’s tau was used to analyze the rank-order correlation of preferences between respondents who taught in the didactic component and those who taught in the clinical component of PA programs. The PI analyzed the responses using independent t-tests for comparing the means of two independent groups to determine if there was a statistically significant difference between them, as well as the Tukey Honestly Significant Difference (HSD) to determine if any differences in the means among different respondents are significant based on years of experience. This study was a mixed-methods, descriptive study.

Ethics statement

This study received approval from Lincoln Memorial University's Institutional Review Board (IRB) prior to data collection (approval number is 797V.0). All participants were adult physician assistant educators voluntarily recruited via a secure and professional listserv. Informed consent was obtained electronically before survey participation, with respondents required to indicate agreement before accessing any research questions. The survey was anonymous, and no personally identifiable information was collected. Participants were informed that they could withdraw from the study at any time without penalty. All data were stored securely and analyzed in aggregate to ensure confidentiality. The study adhered to ethical principles outlined in the Declaration of Helsinki and complied with institutional and federal guidelines for human subjects research.

## Results

The study concluded with 68 valid responses (n = 68). Forty-four (64.7%) of the respondents (PA educators) answered in the affirmative that there is a current deficit in physician assistant student differential diagnosis skills. The respondents who answered in the affirmative were then asked to rate how much of a problem they thought it was. None of the respondents thought the problem to be a minor or critical one. The majority of respondents believed the problem to be a moderate one, 30 (68.2%), six (13.6%) of the respondents thought the issue was a minor problem, and eight (18.2%) thought it to be a significant problem.

The three top solutions included (a) team-based learning events, 10 (23.3%), (b) instructor-led case studies, 10 (23.3%), and (c) incorporate into the current medicine course, 10 (23.3%). However, team-based learning problems had the second highest number of votes for the second-best solution at 12 votes (27.9%). Instructor-led case studies followed the team-based solution for the third place with nine votes (20.9%). There was no statistically significant difference between the clinical and didactic educators’ opinions on the solution (p-value range: 0.18 - 0.99) (Table 1). 

**Table 1:** Independent Samples Two-Tailed T Test (Clinical vs Didactic). This table displays the statistical analysis of solution preferences by education type.

Solution Type	t	df	p	Diff	SEM	95% CI	
						L	U
New Course	0.33	16.90	0.75	0.21	0.64	-1.14	1.55
Independent Study	1.40	24.83	0.18	0.38	0.27	-0.18	0.93
Technology	-1.28	14.53	0.22	-0.68	0.53	-1.81	0.45
Into Medicine Course	0.01	13.65	0.99	0.01	0.67	-1.43	1.44
Team based	0.41	13.74	0.69	0.30	0.73	-1.26	1.86
Small Group (SG)	0.05	14.28	0.96	0.04	0.68	-1.41	1.49
Teacher led SG	-0.49	16.59	0.63	-0.25	0.50	-1.31	0.81

The majority, 23 (51.2%), of respondents reported an already overburdened faculty as the primary etiology of the problem. The second most cited reason was a lack of faculty instruction, 7 (16.3%). The selected reasons why the respondents indicated what solutions they chose for the problem were (a) personal experience, 33 (75.5%), (b) it seems logical, eight (17.6%), and (c) it was an evidence-based decision, three (7.4%). Four (40%) of the clinical physician assistant educators chose their responses based on personal experience, one (10%) based on evidence, and five (50%) stated it seemed logical. The didactic educators chose (a) personal experience, 24 (70.6%), (b) evidence-based decision, three (8.8%), and (c) seems logical, 17 (50%).

The years of the respondents’ experience in physician assistant education varied from 1 to greater than 20 years. The number of respondents with 1 - 5 years of experience was 26 (38.2%), 6 - 10 years of experience was 18 (26.5%), 11 - 20 years of experience was 14 (20.6%), and >20 years of experience was 10 (14.7%). There was no statistically significant difference between years of experience and the solution to the problem (Table 2). None of the comparisons showed statistically significant differences between the clinical and didactic PA educators across the various courses and instructional types.

**Table 2:** Multiple Comparisons (Tukey Honestly Significant Difference) - Changes in Confidence Levels of Physician Assistant Students Post-Intervention. This table displays the comparison of pre- and post-test scores to illustrate the effectiveness of the educational intervention on student confidence in formulating differential diagnoses.

Dependent Variable (Solution to perceived problem)	(I) Years of Experience Group 1	(J) Years of Experience Group 2	Mean Difference (I-J)	Std. Error	Sig.	95% Confidence Interval	
						Lower Bound	Upper Bound
New Course	1 - 5 Yrs	6 - 10 Yrs	0.410	0.729	0.840	-1.36	2.18
		>11 Yrs	0.200	0.702	0.956	-1.51	1.91
	6 - 10 Yrs	1 - 5 Yrs	-0.410	0.729	0.840	-2.18	1.36
		>11 Yrs	-0.210	0.729	0.955	-1.98	1.56
	>11 Yrs	1 - 5 Yrs	-0.200	0.702	0.956	-1.91	1.51
		6 - 10 Yrs	0.210	0.729	0.955	-1.56	1.98
Independent Study	1 - 5 Yrs	6 - 10 Yrs	-0.128	0.367	0.935	-1.02	0.76
		>11 Yrs	-0.400	0.354	0.501	-1.26	0.46
	6 - 10 Yrs	1 - 5 Yrs	0.128	0.367	0.935	-0.76	1.02
		>11 Yrs	-0.272	0.367	0.741	-1.16	0.62
	>11 Yrs	1 - 5 Yrs	0.400	0.354	0.501	-0.46	1.26
		6 - 10 Yrs	0.272	0.367	0.741	-0.62	1.16
Technology	1 - 5 Yrs	6 - 10 Yrs	-0.226	0.557	0.914	-1.58	1.13
		>11 Yrs	-0.533	0.537	0.585	-1.84	0.77
	6 - 10 Yrs	1 - 5 Yrs	0.226	0.557	0.914	-1.13	1.58
		>11 Yrs	-0.308	0.557	0.846	-1.66	1.05
	>11 Yrs	1 - 5 Yrs	0.533	0.537	0.585	-0.77	1.84
		6 - 10 Yrs	0.308	0.557	0.846	-1.05	1.66
Into Medicine Course	1 - 5 Yrs	6 - 10 Yrs	0.072	0.665	0.994	-1.55	1.69
		>11 Yrs	0.333	0.641	0.862	-1.23	1.89
	6 - 10 Yrs	1 - 5 Yrs	-0.072	0.665	0.994	-1.69	1.55
		>11 Yrs	0.262	0.665	0.919	-1.36	1.88
	>11 Yrs	1 - 5 Yrs	-0.333	0.641	0.862	-1.89	1.23
		6 - 10 Yrs	-0.262	0.665	0.919	-1.88	1.36
Team based	1 - 5 Yrs	6 - 10 Yrs	0.103	0.721	0.989	-1.65	1.86
		>11 Yrs	0.667	0.695	0.607	-1.03	2.36
	6 - 10 Yrs	1 - 5 Yrs	-0.103	0.721	0.989	-1.86	1.65
		>11 Yrs	0.564	0.721	0.716	-1.19	2.32
	>11 Yrs	1 - 5 Yrs	-0.667	0.695	0.607	-2.36	1.03
		6 - 10 Yrs	-0.564	0.721	0.716	-2.32	1.19
Small Group	1 - 5 Yrs	6 - 10 Yrs	0.246	0.696	0.934	-1.45	1.94
		>11 Yrs	-0.133	0.671	0.978	-1.77	1.50
	6 - 10 Yrs	1 - 5 Yrs	-0.246	0.696	0.934	-1.94	1.45
		>11 Yrs	-0.379	0.696	0.850	-2.07	1.32
	>11 Yrs	1 - 5 Yrs.	0.133	0.671	0.978	-1.50	1.77
		6 - 10 Yrs.	0.379	0.696	0.850	-1.32	2.07
Teacher-led small group	1 - 5 Yrs.	6 - 10 Yrs.	-0.477	0.567	0.680	-1.86	0.90
		>11 Yrs.	-0.133	0.546	0.968	-1.46	1.20
	6 - 10 Yrs.	1 - 5 Yrs.	0.477	0.567	0.680	-.90	1.86
		>11 Yrs.	0.344	0.567	0.818	-1.04	1.72
	>11 Yrs.	1 - 5 Yrs.	0.133	0.546	0.968	-1.20	1.46
		6 - 10 Yrs.	-0.344	0.567	0.818	-1.72	1.04

Table 2 presents the results of a Tukey HSD test, which compared the data of different groups to see if they are significantly different from each other based on their years of experience in PA education. We used different types of courses or learning methods (i.e., new courses, independent study, into medicine course, etc.), but there were no significant differences in the mean scores between the groups based on years of experience. This means that the amount of experience (whether 1-5 years, 6-10 years, or more than 11 years) does not significantly affect the scores for this survey.

## Discussion

Most, 44 (64.7%), physician assistant educators believe there is a current deficit in PA students' differential diagnosis skills. which underscores the significant gaps identified in previous research [1,5]. This deficit not only impacts the clinical effectiveness of future PAs but also stresses the urgent need for curriculum reforms that emphasize problem-solving and clinical reasoning.

The respondents were divided on the solution to this deficit. However, two of the three solutions are closely related as a type of small group - small groups with team-based learning problems and instructor-led case studies. Team-based learning problems were the second choice of the majority at 12 (27.9%). This indicates the educators believe the answer lies in problem-based learning modalities. Incorporating differential diagnosis content into the current medical course was tied for second place as a solution, 10 (23.3%). Intelligent differential diagnosis requires critical thinking, which may be difficult to incorporate into a medicine course, particularly if it is lecture-based and is assessed with only multiple-choice examinations.

Educators' preference for team-based learning, 10 (23.3%), and instructor-led case studies, 10 (23.3%), as solutions reflects a growing consensus that active and engaged learning strategies can enhance diagnostic competencies [4]. These methods align with findings by Chew et al., who noted that checklists and structured learning tools significantly boost the confidence and accuracy of medical students in making differential diagnoses [3].

Interestingly, integrating differential diagnosis into existing medical courses, though viewed as beneficial by 10 (23.3%) respondents, presents challenges. The traditional lecture-based format may not provide sufficient interaction and practical experience necessary for mastering such a complex skill. This supports the argument by Alexander et al., who advocate for a more hands-on approach in clinical education to bridge the gap between theoretical knowledge and practical application [2].

Furthermore, the split opinion among educators about the best approach to teaching differential diagnosis highlights the need for diverse educational strategies to cater to different learning environments, learning styles, and student needs. Future curricula might benefit from a hybrid model that incorporates traditional lectures, problem-based learning, and digital tools, as suggested by the emerging trends in educational technology.

A multi-institutional study used simulation to teach cardiopulmonary physical examination and diagnosis skills, highlighting the effectiveness of hands-on learning environments [6]. Another study explored the benefits of reflection as a strategy to enhance medical students' diagnostic competencies, suggesting that structured reflection on clinical cases improves diagnostic skills [6,7]. These studies emphasize the importance of active learning and critical thinking in developing diagnostic competence.

The use of simulation-based training, as explored by Multak et al., effectively enhances cardiopulmonary examination and diagnostic skills in PA students, suggesting that similar approaches could be beneficial for teaching differential diagnosis [6]. Moreover, Mamede et al. demonstrate that reflective practice can significantly improve the diagnostic competencies of medical students, supporting its inclusion in PA training programs to foster a deeper understanding and retention of diagnostic skills [7].

Educators' preference for team-based learning and instructor-led case studies reflects a growing consensus that active learning strategies can substantially enhance diagnostic competencies. These methods, complemented by structured reflective exercises, could address the identified educational gaps more effectively than traditional lecture-based formats, which may not provide sufficient interaction and practical experience necessary for mastering such complex skills. To ensure the development of effective differential diagnosis skills, PA programs should consider these varied instructional strategies, which have been shown to enhance critical thinking and clinical decision-making in a range of healthcare professions [3,4]. This research and related studies suggest that adopting a multimodal approach, integrating both problem-based learning and traditional lectures, offers the most effective solution.

There are many ways in which to incorporate more differential diagnostic learning and practice. A mnemonic-based checklist was used successfully by a university in Malaysia (Universiti Sains Malaysia) to improve their medical students’ differential diagnosis skills. The mnemonic checklist also helped the student avoid closing the medical cases too soon. The first step in differential diagnosis development typically occurs after the patient interview and is called script activation. Scripts are restored, structured networks of knowledge that are activated in working memory. Then script evaluation is driven by the collection of more patient data until the signs and symptoms match the most probable or working diagnosis [3]. 

Differential diagnosis proficiency requires extensive medical knowledge, with the ability to fall back on the basic sciences when struggling to develop differentials. Not assessed in the study was what role students’ knowledge of the basic sciences may have played in their differential diagnosis formulation. Using advanced anatomy knowledge can help expand the differential diagnosis list based on the location of a problem - knowledge of physiology and pathophysiology is necessary to understand patient symptomatology. There are several differential diagnosis training software programs available now. Future research is needed to evaluate PA students’ mastery of the basic sciences, as well as the analysis of differential diagnosis software incorporation into PA education programs. Future research should examine the long-term impact of team-based learning on diagnostic accuracy. Additionally, studies should investigate the ideal curriculum structure for integrating differential diagnosis training into physician assistant education. Further exploration of student perceptions regarding effective differential diagnosis teaching strategies is also warranted.

Limitations of the study include having a small sample size. Although the survey took the average respondent 2 minutes and 12 seconds, the busy schedule of the educators may have played a role in the small sample size (e.g., non-response bias). Regardless of the paucity of research on the topic, the brevity of the study, and the ease of access, the survey response appeared to be the greatest limitation in the author’s research efforts. The survey study is difficult to authenticate, however, the PA Education Association (PAEA) listserv is private, and only PAEA personnel are authorized to put emails on the listserv. Non-response bias was the most significant limitation. Although the survey was disseminated through the PAEA listserv to a broad national sample, the use of convenience sampling may limit the generalizability of the results. Faculty who chose to participate may differ meaningfully from those who did not, and the absence of a reported response rate prevents accurate assessment of representativeness. Despite these limitations, the findings offer valuable preliminary insights into perceptions of differential diagnosis proficiency among PA students and potential strategies for curriculum development. As with all survey-based research, the reliance on self-reported data introduces the potential for response bias. Participants may have answered questions in a manner that reflects their aspirations for their programs or students, rather than objective assessments. Additionally, although the survey instrument was pilot tested and peer-validated, it did not undergo formal psychometric evaluation. Therefore, the reliability and validity of the instrument have not been empirically established, which may impact the consistency of findings across different educational contexts. While the study incorporated open-ended responses to capture qualitative perspectives, a formal thematic or content analysis was not performed. As such, the qualitative data were not analyzed with the same level of methodological rigor as the quantitative data, which may have limited the depth and interpretability of respondents' narrative insights.

## Conclusions

This study found that the majority of respondents (44 respondents; 64.7%) affirmed the existence of a deficit in physician assistant (PA) students’ differential diagnosis skills. Among these respondents, most (30 respondents; 68.2%) characterized the gap as a moderate problem, whereas fewer described it as minor (six respondents; 13.6%) or significant (eight respondents; 18.2%). To address this gap, participants equally endorsed three primary solutions: team-based learning events, instructor-led case studies, and incorporating differential diagnosis training into the existing medicine course (each selected by 10 respondents; 23.3%). Notably, team-based learning also received strong support as a secondary approach (12 respondents; 27.9%), while independent study was overwhelmingly ranked as the least effective solution (30 respondents; 69.8%).

These findings highlight the need to strengthen differential diagnosis instruction in PA education and can inform immediate curricular enhancements. It is recommended that all identified strategies be implemented in combination, ideally within a competency-based medical education framework. Organizing the curriculum into organ system-based or specialty-based blocks with horizontal integration may improve coherence and reinforce diagnostic reasoning skills. Future research should validate these results in larger, more diverse samples to improve generalizability. Studies are also needed to evaluate the effectiveness of the proposed instructional strategies (e.g., team-based learning, case studies, and curricular integration) in improving diagnostic proficiency, preferably using longitudinal or experimental designs to track skill development over time. 

## Figures and Tables

**Figure 1:**
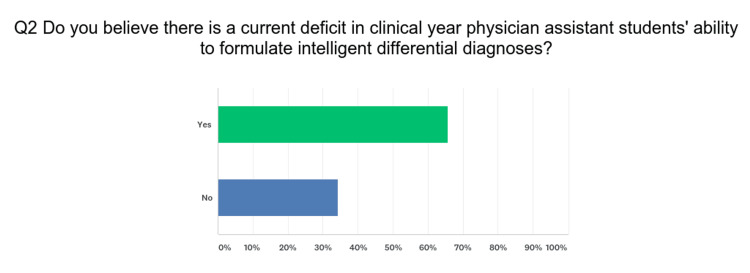
Question 2 of Survey

**Figure 2:**
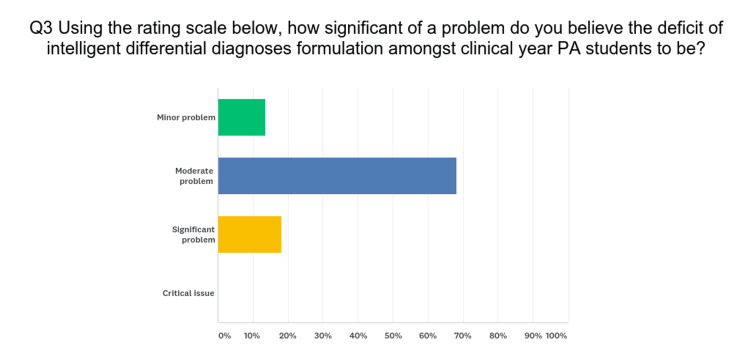
Question 3 of Survey

**Figure 3:**
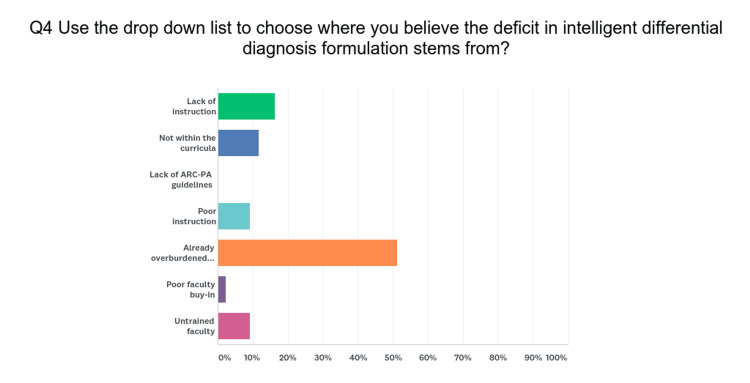
Question 4 of Survey

**Figure 4:**
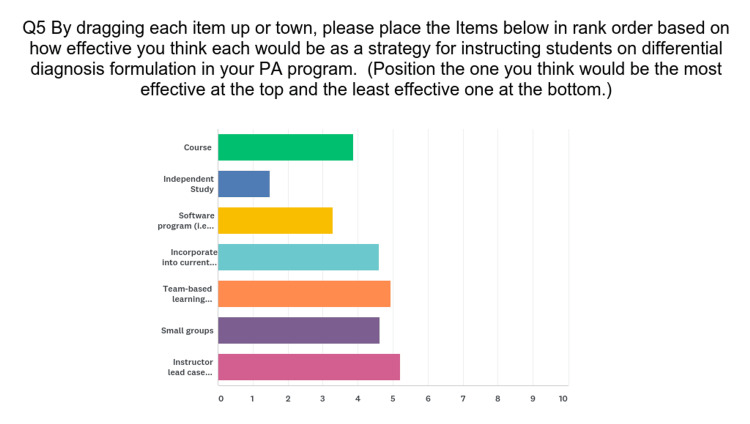
Question 5 from Survey

**Figure 5:**
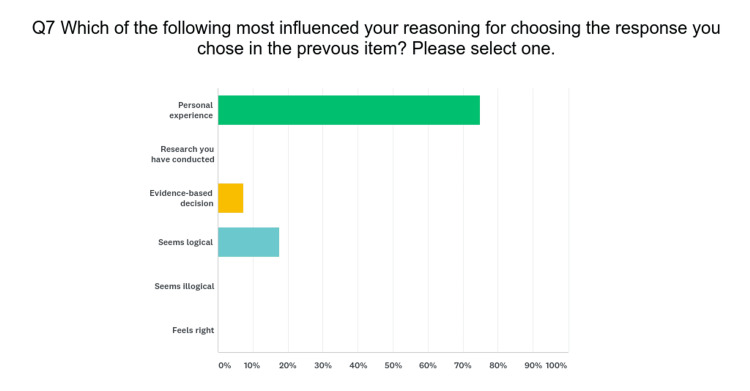
Question 7 of Survey

**Figure 6:**
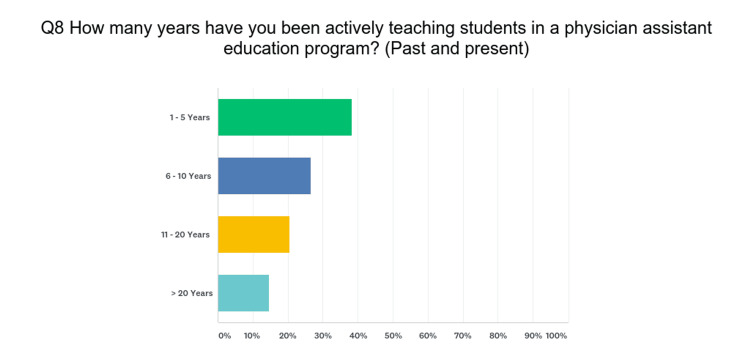
Question 8 from Survey

**Figure 7:**
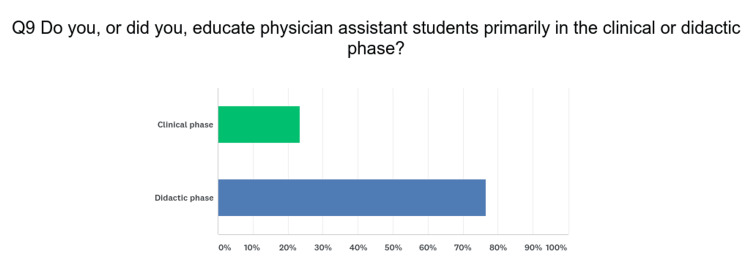
Question 9 from Survey
